# ASSESSMENT OF VISUAL PROBLEMS AFTER ACQUIRED BRAIN INJURY: A SURVEY OF CURRENT PRACTICE IN DANISH HOSPITALS

**DOI:** 10.2340/jrm.v56.28793

**Published:** 2024-05-14

**Authors:** Trine SCHOW, Eike Ines WEHLING, Helle K. FALKENBERG, Anne NORUP, Karin Spangsberg KRISTENSEN

**Affiliations:** 1 Neurorehabilitation Research and Knowledge Centre, Rigshospitalet, Copenhagen, Denmark; 2 Neurorehabilitation-Cph, Copenhagen, Denmark; 3 Department of Biological and Medical Psychology, University of Bergen, Bergen, Norway; 4 Department of Physical Medicine and Rehabilitation, Haukeland University Hospital, Bergen, Norway; 5 Department of Optometry, Radiography and Lighting Design, University of South-Eastern Norway, Notodden, Norway; 6 Department of Neuroscience, University of Copenhagen, Copenhagen, Denmark

**Keywords:** brain injuries, stroke, surveys and questionnaires, symptom assessment, vision disorders

## Abstract

**Objectives:**

To explore current hospital practice in relation to the assessment of vision problems in patients with acquired brain injury.

**Design:**

A survey study.

**Subjects:**

A total of 143 respondents from hospital settings, with background in occupational therapy and physical therapy, participated in the survey.

**Methods:**

The survey questionnaire, developed collaboratively by Danish and Norwegian research groups, encompassed 22 items categorically covering “Background information”, “Clinical experience and current practice”, “Vision assessment tools and protocols”, and “Assessment barriers”. It was sent out online, to 29 different hospital departments and 18 separate units for occupational therapists and physiotherapists treating patients with acquired brain injury.

**Results:**

Most respondents worked in acute or subacute hospital settings. Few departments had an interdisciplinary vision team, and very few therapists had formal education in visual problems after acquired brain injury. Visual assessment practices varied, and there was limited use of standardized tests. Barriers to identifying visual problems included patient-related challenges, knowledge gaps, and resource limitations.

**Conclusion:**

The study emphasized the need for enhanced interdisciplinary collaboration, formal education, and standardized assessments to address visual problems after acquired brain injury. Overcoming these challenges may improve identification and management, ultimately contributing to better patient care and outcomes in the future.

LAY ABSTRACT

Vision problems are common after acquired brain injury, but no clear national guideline in Denmark exists in relation to assessing vision problems. This study looked at how therapists identify vision problems in patients who have acquired a brain injury. We surveyed 143 therapists working in hospitals. Interestingly, only a few had a practical guideline for how to do the assessment. Moreover, very few worked in teams, and therapists did not have much formal training on these problems. Therapists had different ways of assessing patients’ vision, and some faced challenges like not having enough knowledge or resources. The study suggests that better interdisciplinary teamwork is needed, more education, and common ways of checking vision problems after a brain injury to improve how we treat patients with vision problems in Denmark.

Visual problems following acquired brain injury (ABI) are a complex and multifaceted condition (1) that poses significant challenges to individuals (2), healthcare professionals, and society (3–6). Various aspects of visual processing lead to a wide range of visual impairments (7), including reduced visual acuity, visual field defects, oculomotor dysfunction, binocular vision abnormalities, and higher-level visual processing deficits (8). The prevalence of overall visual problems after ABI has been estimated to range from 54% to 73% (9, 10), depending on the type of problem, time of assessment, and assessment methods used. The consequences of visual problems interfere with the ability to participate in rehabilitation, daily activities, and social events. In turn, quality of life and general rehabilitation of the person is reduced (11–15).

Some visual problems are easily detected as part of the general medical/neurological examination, while others are not. Asking the patient about perceived problems has shown that unawareness of these problems is common, and they may not experience complaints of any visual symptoms at all (2, 16, 17). Therefore, comprehensive assessment as early as possible is essential to accurately diagnose and manage visual problems. Due to their complex impact, an interdisciplinary approach has been suggested to be preferable, comprising specialists from the field of neurology, ophthalmology, optometry, and rehabilitation (18). So far, there is no gold standard or interdisciplinary clinical guideline for how to assess visual problems in hospital settings, and the use of screening tools and assessment procedures varies between hospitals and countries. In a review, Hanna and colleagues (19) found that no single tool may be used to screen for all potential visual impairments after stroke. Only a few studies have investigated current practice within visual assessment in nationwide studies. These either focused on the role of one specific profession such as occupational therapists(20), or on one specific visual problem (21, 22).

In Denmark, a neurologist or physician is responsible for initial screening and diagnosing visual problems. The general neurological examination includes an examination of the cranial nerves, eye movements and visual fields, neuro-ophthalmic reflexes, and inattention. However, other professions, mostly occupational therapists (OT) and physiotherapists (PT), are involved when identifying the impact of different visual problems on the patients’ activities, and participation in and observation of undetected visual problems in hospital settings. Neuropsychologists are primarily responsible for perceptual problems and neglect. The Danish health authority recommends that professionals with specific knowledge of visual problems should assess patients with ABI when there is a suspicion that the patient might have visual problems (23). So far, there is no specific clinical guideline for when and how other professions may be involved in the assessment of visual problems in Denmark and different practices are very likely employed across hospitals. To the best of our knowledge, no previous studies have investigated the broader concept of visual assessment and its practice in hospital settings in a Danish context. Thus, the purpose of this study was to investigate current practice in relation to the assessment of visual problems after acquired brain injury in a national survey of Danish hospitals and healthcare professions.

## METHODS

This paper reports the results of a national survey study on assessment of visual problems in Danish hospitals. The study is part of a larger Nordic survey. This survey assesses the current clinical practice of assessment, identification, protocols, and referrals of visual problems after brain injury. It is being conducted in collaboration with the Norwegian research group. The results from the Norwegian study will be published separately. The survey was conducted as an anonymous, online analysis using the Research Electronic Data Capture (REDCap^®^) system (24).

### Survey design

An interdisciplinary group of researchers and clinicians from Danish and Norwegian hospitals and universities developed the questionnaires. The project group was inspired by earlier surveys (20–22) and involved counselling from national and international vision experts. The survey was designed to gather information on clinical practice regarding identification of visual problems across different professions and hospital departments involved in the treatment of patients with ABI.

### Survey construct

The survey was introduced with operational definitions:

- Acquired brain injury: Stroke, TBI (except concussion), brain tumour, infections (except COVID-19), sepsis, anoxic damage, or substance abuse.
- Identification of visual problems: Any method (observation, screening, or assessment) with the purpose of gathering identification of potential visual problems following ABI.
- Visual problems were defined as:
  - Sensory vision problems: Damage to the early nerve pathways between the eye and the primary visual and motor cortex.
  - Visual perception refers to the processing of visual information that lies beyond the primary cortex.
  - Visual neglect/inattention refers to reduced awareness of visual stimuli.

Core items comprised “*Background information*” including type of profession, level of experience, type of unit and patients, years of experience; “*Clinical experience and current practice*”: addressing questions regarding workplace, years of experience with visual problems and more; “*Vision assessment tools and protocols*” including kind of visual assessment tools used;§ and a section concerning “*Assessments barriers*” relating to patient characteristics, staff resources, facilities, and materials.

### Pilot of the survey

A Danish pilot survey was distributed in 2 different hospital departments, involving 12 respondents within 5 different professions, together with an evaluation questionnaire addressing survey construct, grammar, design, and missing or redundant questions. Eleven professionals responded to the survey design. One nurse did not complete the visual assessment survey but only the evaluation questionnaire, as nurses in her department never conducted visual screening or assessment. Another nurse did respond to the questionnaire but reported in a similar way, i.e. that she and her colleagues never conducted vision screening or assessment. As this information was consistent with our clinical experience, it was decided to exclude nurses from the survey. Other comments and suggestions were incorporated, and the final survey consisted of 22 items. The survey covered four topics: (*i*) clinical practice (e.g., routines, teamwork), (*ii*) assessment methods, (*iii*) assessment barriers and (*iv*) background information including profession, clinical experience, and type of workplace.

### Data collection

The survey questionnaire was sent out in February and March 2023. Prior to the survey, all Danish hospitals treating patients with ABI were contacted. This included neurosurgery units, acute neurology, plus acute and subacute neurorehabilitation. It also included non-specialized departments at smaller hospitals, where ABI patients may be hospitalized and treated in departments together with other diagnoses. We identified a total of 29 departments with neurological patients in Denmark and 18 separate units for occupational therapists (OTs) and physiotherapists (PTs). At some hospitals OTs/PTs are organised in separate units and in other hospitals they are employed directly on the neurological ward. We recruited by reaching out to the head of the department or head of unit requesting their consent to participate and to identify a project contact who was contacted for further distribution. Upon receiving acceptance, a survey link was sent to the designated project contact within the department for further distribution.

**Fig. 1:**
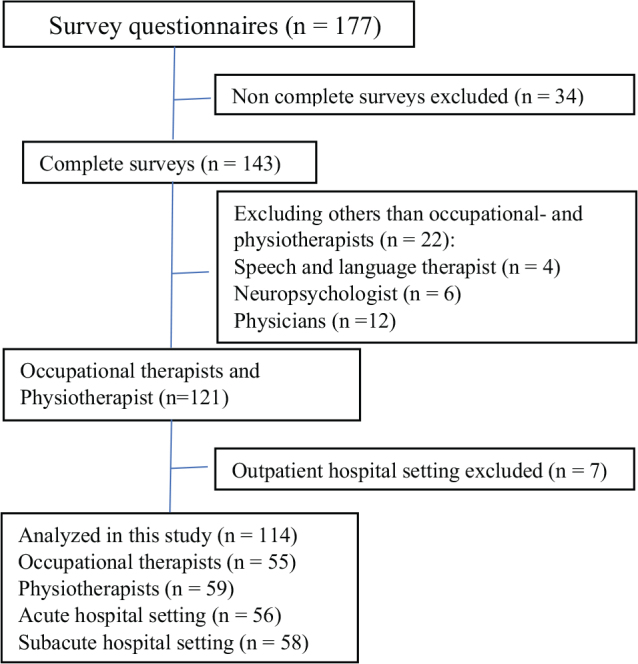
Survey methodology process.

Potential respondents received identical links to ensure anonymity. No personally sensitive information or IP addresses were collected. According to the Danish ethical regulations, no ethical approval was required for the present study. The project contacts were asked to report how many staff members received the link. We re-invited departments after 1 month if no response was obtained. Further reminders were sent close to the survey deadline (see Fig. 1). Of the 29 departments with neurological patients and 18 separate OT/PT units, 12 ABI wards and 11 OT/PT units agreed to participate. Six ABI wards did not wish to participate, 3 wards no longer treated ABI patients, and 1 unit with OT/PTs did not wish to participate. Eight wards never replied to the invitation.

The study was approved by the Regional Danish data authority J-nr: P-2022-457.

### Statistical analysis

Only complete surveys were included in the analysis. There were very few surveys obtained from respondents without an OT or PT background (15%). Due to the risk that these responses were not representative of their profession at large, these were excluded from further analysis.

Descriptive statistics were calculated for categorical and continuous responses and are presented as mean for continues variables and median for categorical, frequencies, and percentages. Mean differences between groups were calculated using the Mann–Whitney *U* test (2-tailed). A significance level of 0.05 was used. All data were analysed using SPSS^®^ Version 29.0.1.0. for Windows^®^ (IBM Corp, Armonk, NY, USA) (25).

## RESULTS

A total of 177 surveys were opened and 143 were complete (Fig. 1). After excluding respondents without an OT or PT background, a total of 121 surveys remained.

Only 7 respondents (6%) worked in an outpatient hospital setting. These and they were excluded from the rest of the analysis, as their clinical practice differed substantially from in-hospital settings. A total of 114 respondents are included in further analysis.

### Clinical practice

As seen in Table I, OTs comprised 48% and PTs 52%. Most PTs (66%) worked in acute care, and most OTs (62%) worked in subacute care. The majority of respondents worked with more than 1 type of patient (55%) or with stroke patients exclusively (44%). Most respondents examined only 0–1 patients per week (42%) or 2–5 patients per week (36.8%). About 50% examined all patients with ABI for visual problems and the other half examined only on suspicion of visual problems.

**Table I:** Clinical background, clinical setting, and clinical experience

Factor	*n* (%) *
Profession, *n* (%)	
OT	55 (48)
PT	59 (52)
Hospital setting, *n* (%)	
Acute	56 (49); OT = 34, PT = 66
Subacute	58 (51); OT = 62, PT = 38
Number of beds for ABI patients at the ward, mean (SD)	20 (10)
Median (range)	20 (2–80)
Number of days from hospitalization to assessment of VP, mean (SD)	3 (4.5)
Median (range)	2 (0–21)
Number of patients examined for VP each week, *n* (%)	
0–1	48 (42)
2–5	42 (37)
5–10	15 (13)
> 10	9 (8)
Years of experience, mean (SD)	8 (8)
Median (range)	9 (0–37)
Professional level, *n* (%)	
Novice	12 (11)
Advanced beginner	49 (43)
Competent	38 (33)
Proficient	14 (12)
Expert	1 (1)
Type of patients, *n* (%)	
Stroke	50 (44)
TBI	0 (0)
Other patients	1 (1)
More than one type of patients	63 (55)

OT: Occupational therapist; PT: Physiotherapist; SD: standard deviation.

Respondents reported a mean number of 20 (SD: 10) beds with neurological patients on their ward and the average number of days from hospitalization to visual assessment was 3 (SD: 5). Respondents had a mean of 10 (SD: 8) years of experience identifying visual problems, and most respondents rated themselves as novice or advanced beginners (54%). Only 1 reported being an expert. Achievement of qualifications was primarily through own experience (*n* = 63) or consulting colleagues (*n* = 110). Only 1 person had a formal education.

In total, 23% had an interdisciplinary vision team in their clinical setting. Team members were OTs (18%), PTs (21%), and neuropsychologists (14%). Very few reported that a physician (5%), a speech and language therapist (2%), or other professions (1%) was part of the vision team.

Respondents were asked whether their department had an instruction or guideline that included identification of visual problems, 36% confirmed this, 21% answered that it was part of the general neurological examination and 23% reported that they had no guideline and 20% reported not knowing.

### Visual assessment/screening

**Fig. 2:**
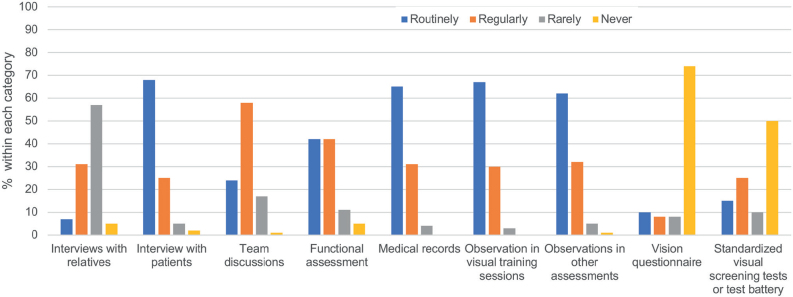
Reported use of different clinical assessment methods. Routinely: with all patients; Regularly: with selected patients; Rarely: with some patients; Never: with no patients.

The respondents reported using both standard and non-standard assessments and tests to identify vision problems. An overview and characteristics of the methods can be seen in Fig. 2 and Table II. Fig. 2 shows that the majority (82%) never or rarely used vision questionnaires, and 60% never or rarely used standardized visual screening tests. The most routinely used methods were patient interviews (68%), medical records (65%), observation in visual training sessions (67%), or observations in other assessments (62%). Team discussions were also used regularly (58%).

**Table II:** Reported use of standardized tests and questionnaires

Factor	Yes Physiotherapist *n* (%)	Yes Occupational therapist *n* (%)
Standardized tests		
Cortical Vision Screening Test (CORVIST)	2 (3)	2 (4)
Donders’ test	7 (12)	2 (4)
H-test	**31 (53)**	**8 (14)**
Motor free visual perception test	1 (2)	0 (0)
Perimetry	3 (5)	0 (0)
Vestibulo-ocular reflex (VOR)	**14 (24)**	**2 (4)**
Visual Object and Space Perception Battery (VOSP)	1 (2)	2 (4)
Other	2 (3)	8 (15)
Questionnaires		
Cerebral vision questionnaire	0 (0)	0 (0)
Convergence insufficiency symptom score	0 (0)	0 (0)
Self-reported Assessment of Functional Visual Performance	0 (0)	0 (0)
The national Eye Institute Visual Function Questionnaire	0 (0)	0 (0)
The Visual Interview	0 (0)	0 (0)
Visual Activity Questionnaire	1 (2)	0 (0)
Self-made questionnaire	9 (15)	8 (15)
Other	**3 (5)**	**10 (18)**

*p* < 0.05 marked in bold.

The 40% of respondents who reported using standardized vision tests or assessments routinely or regularly were asked which tests they used. The most frequently reported were the eye movement test Vestibular Ocular Reflex test (VOR) (26) (27%) and the H-test (57%). Significantly more PTs used both the H-test (27) (*p* = < 0.001) and the VOR test (*p* = 0.002) more often than OTs. Only 18% responded that they used vision questionnaires, which were almost exclusively constructed by themselves or colleagues (see Table II for details).

### Observation in activities

Significant differences were found regarding observation of activities that could lead to identification of visual problems. More OTs used instrumental daily activities (*p* < 0.001), Kessler Foundation Neglect Assessment Process (KF-NAP™) (*p* < 0.001) and primary daily activities (*p* < 0.001) than PTs. PTs significantly used more observations of mobility and transfer activities to identify visual problems (*p* < 0.001) (see Table III).

**Table III:** Reported use of activities with the purpose of identifying visual problems

Factor	Mean ranks* PT	Mean ranks* OT	*p*-value
Instrumental activities of daily living, e.g. cooking or cleaning	38	79	< 0.001
Kessler Foundation Neglect Assessment Process (KF-NAP™)	49	66	< 0.001
Reading	53	62	0.124
Mobility and transfers	68	46	< 0.001
Basic activities of daily living, e.g. dressing or eating	48	68	< 0.001
Safety in traffic, e.g. crossing the street	58	57	0.915
Other	37	33	0.167

* Low mean rank score corresponds to lower use of the corresponding test.

OT: Cccupational Therapist; PT: Physio Therapist.

### Assessing specific visual problems

**Fig. 3:**
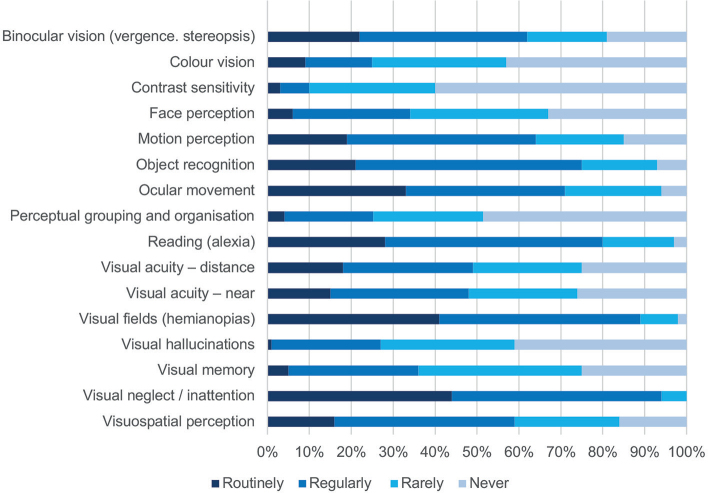
Assessment of different kinds of visual problems.

Fig. 3 presents the reported frequency of assessment of different types of visual problems.

Respondents reported that they assess many different visual functions. The most frequently routinely assessed were visual neglect, visual fields, ocular movements, and reading, whereas contrast sensitivity, perceptual grouping, colour vision, and visual hallucinations were most commonly never assessed (41–60%).

### Referral to vision specialists

**Fig. 4:**
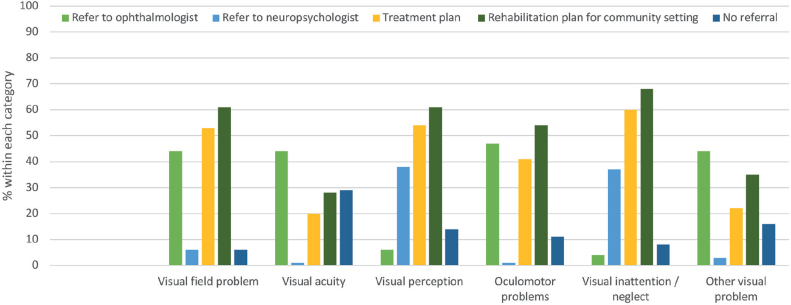
Referral of different types of visual problems.

Fig. 4 shows the respondents’ knowledge of specific referral procedures for some of the main categories of visual problems. Respondents could select all responses that applied. Approximately 45% referred visual field problems, reduced visual acuity, oculomotor problems, and other visual problems to an ophthalmologist. For visual perception problems and visual neglect, most (40%) referred to a neuropsychologist. Many respondents did not know where to refer a patient with reduced visual acuity (29%), visual perception problems (14%), oculomotor problems (11%), or other non-specific vision problems (16%).

### Aim in identifying visual problems

Most respondents 50% used the visual assessment results in the in-hospital treatment plan, except from visual acuity, which was used by only 20%. Similarly, about 60% would use the information to provide a rehabilitation plan for a community setting, again except for visual acuity, which was used by only 28%.

### Barriers

Respondents were asked about certain barriers and challenges to identifying vision problems in their patients, and answered all that applied. Multiple choices were possible.

**Fig. 5:**
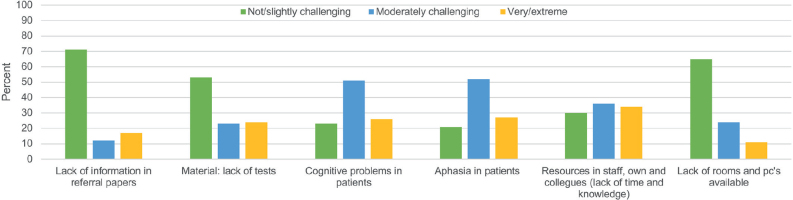
Barriers to the assessment of visual problems. Multiple-choice answers were possible. Bars presents number of responses.

The main barriers were lack of time and knowledge; patients’ ability to participate due to cognitive or language problems were rated as moderate barriers. The 3 least challenging categories were lack of information in referral papers, or test materials, and physical resources (see Fig. 5).

## DISCUSSION

Our survey aimed to explore the current practice of occupational therapists (OTs) and physiotherapists (PTs) assessing visual problems in departments treating acquired brain injury (ABI) patients. The results showed that despite being heavily involved in the identification of visual problems, these professions used very few standardized tests and had no formal education in the assessment of visual problems. Further, very few departments had a specific practice guideline or structured routine to support their clinical vision assessment practice.

The survey was distributed in hospital departments with neurological patients, to several different healthcare professionals involved in visual assessment. However, respondents were almost all OTs and PTs. This outcome is similar to another Danish survey focusing on the assessment of spatial neglect, where the predominant respondents were also OTs (48%) and PTs (17%) (22). This response pattern might highlight the significant role played by OTs and PTs in the assessment of visual problems in Danish hospitals. The lower response rates of other professions may have varying reasons that cannot be definitively determined. One reason could be that physicians, who are primarily responsible for vision assessment in Danish hospitals, have limited time to prioritize participating in surveys. Other professions such as speech and language therapists or neuropsychologists may mostly be interested in single functions and activities like reading or visual perception and therefore may not be comfortable participating in an extensive assessment of visual function.

Importantly, OTs and PTs do not have a formal role or clinical expertise in visual assessment, and none of the respondents reported having an ophthalmologist or other vision expert in the interdisciplinary team. This is a concern, as involvement of vision experts in the hospital departments or as a support service would improve the identification and follow-up of visual problems, as has been suggested by a consensus study of the vision care pathway in the UK by Rowe and colleagues (28).

### Care settings and clinical practice

Despite the considerable experience of the OTs and PTs in our survey, most had almost a decade of working experience with visual problems in neurological patients. However, more than half rated themselves as novices or advanced beginners. An explanation for this could be that most of the respondents examined less than 5 patients per week, which may not be enough practice to provide confidence in their own skills and knowledge. Moreover, the lack of focus on vision in OTs’ and PTs’ professional education programmes and few respondents who had undertaken continuing educational courses may contribute to understanding these findings. Our findings are similar to those by Pollock et al. (20), where 24% of OTs reported not having received specific training with regard to visual problems after stroke. They found that knowledge and skills in assessing visual problems were learned from colleagues and their own experience. This supports the need for improved structured clinical practice, for example a systematic clinical guideline for the assessment of visual problems. In our study, respondents also lacked formal education and most rated themselves as novices or advanced beginners despite having many years of clinical practice. This suggests that vision assessment should be included in the formal education of healthcare professionals, including OTs and PTs.

### Visual assessment/screening

When asked about assessment methods, respondents primarily reported utilizing patient interviews, medical records, functional assessments, and observations. Remarkably, only 15–25% used standardized tests routinely or regularly. This is below half of all respondents. Of these, measuring eye movements (H-test) and the vestibulo-ocular reflex test were most frequently used. These tests assess important but quite limited aspects of visual function. Contrary to our results, Rowe (29) reported significant use of standardized assessment in international orthoptic practice. This may serve as an inspiration to the OTs’ and PTs’ clinical practice, and to develop a standardized core set of tests that could be used for all patients, including novice staff.

Our findings indicate further that only about one-third of the respondents had a specific guideline for visual assessment. These working in departments without specific guidelines may be challenged in their clinical practice. This is supported by the finding that more than half rated themselves as advanced beginners or novices. Moreover, the absence of clear guidelines, combined with the limited use of standardized tests, suggests the need for a well-defined protocol and/or flowchart detailing who should assess specific impairments using specific standardised tools, questionnaires, or observation methods. The combination of low level of experience and lack of clinical guidelines may result in inconsistencies in patient management across hospital settings. Yet this issue seems not unique to Denmark: Pollock et al. showed that less than 1 in 10 Scottish hospitals had a guideline for visual problems assessment in Scottish hospitals (20).

It is a concern that only half reported routinely assessing all patients for visual problems, with the remaining conducting vision assessments only upon suspicion. This is a problem, as 40% of stroke patients do not report or articulate visual problems or symptoms in the acute phase (19), even if a number of guidelines recommend either that all stroke patients should be offered a vision specialist assessment before leaving the hospital, or an urgent outpatient appointment (31) using a standardized approach (32). To facilitate appropriate referrals for further vision services, vision rehabilitation, and follow-up it is imperative that all patients are examined systematically with the purpose of identification of visual problems (13, 32).

### Referral

Rowe and colleagues (33) developed a stroke-vision pathway to outline ways through which stroke survivors with visual problems can access healthcare services including the appropriate referral(s) relevant to their specific problems. They suggest that problems related to eye position, eye movements, and/or visual fields are referred to orthoptists, visual acuity problems to an optometrist, ophthalmologist, or to the low-vision service, and visual inattention to a stroke team including occupational therapists, with the added option of referrals to orthoptists and/or neuropsychologists when appropriate. It is important to note that not all countries, including Denmark, must find a pathway that is within the context of the national health services. For example, Denmark does not have orthoptists or similar professions within hospital settings. However, there is a need for vision specialists to be included, as our data revealed that only 44% and 47% of respondents refer patients regularly to an ophthalmologist if they have visual field or eye movement assessments, respectively. As such, a referral practice could be reconsidered, and also the utilisation of optometrists. The National Stroke Guideline (32) recommends that “People with altered vision, visual field defects or eye movement disorders after stroke should receive information, support and advice from an orthoptist and/or an ophthalmologist”. As there are no Danish clinical guidelines within visual assessment, it seems evident that clinical practice could be inspired by the work of NICE (34) and the National Clinical Guideline for Stroke for the UK and Ireland (32). Moreover, Rowe and colleagues documented that even though bedside visual screening was possible at a median of 3 days post stroke (35), only 7% of stroke units in the UK had a policy relating to vision assessment (20). Based on those results, Rowe developed a core outcome set for vision screening comprising 9 domains and a full vision assessment comprising 11 domains (36). These guidelines may also serve as an inspiration for developing guidelines in Denmark.

### Barriers

The most significant barriers identified in our survey were lack of time and knowledge, followed by patients’ cognitive and communicative problems. Correspondingly, a Norwegian study from 2021, investigating barriers to the implementation of structured visual assessment after stroke in municipal healthcare services, found some of the same barriers such as time constraints and lack of staff experience (37). These results clearly demonstrate the need for more formal education among OTs and PTs in Danish practice. Communication problems and cognitive problems are known factors involved in the assessment for visual problems (38). This also underscores the critical need for standardization and training in visual problems assessment for PTs and OTs.

### Study strengths and limitations

A strength of this study is that it is the first large study investigation of assessment of a broad category of visual function in Danish hospitals. It has provided a valuable insight into the Danish healthcare system in relation to vision assessment and points to some areas for improvement, specifically the need for training in the assessment of visual function, formal education, and clinical guidelines.

There are limitations to the present study. First, departments accepting to participate in this survey might have a special interest in visual problems and recognize that this area needs more attention. However, hospital departments where there is a well-functioning visual assessment procedure might not have any interest in responding to a survey.

We distributed this survey to a multidisciplinary group of professionals and had an overrepresentation of OTs and PTs. Consequently, other questions might have been included if we had known beforehand that we were investigating those 2 professions exclusively. Finally, this study has been conducted in Denmark, in the context of the Danish healthcare model, and does not represent other healthcare systems.

### Perspectives

This survey highlights a pressing need for more comprehensive training and competencies in standardized assessment methods to ensure the effective identification and management of visual problems in hospital settings. The survey underscores the critical need for the coordination and structuring of the assessment of visual problems in Danish hospitals. The field could benefit from a clinical guideline for all healthcare professions specifying responsibilities, and outlining who should assess visual problems, how, and when. This could be accompanied by educational courses to support clinical expertise. This work could be directed by the Danish Health Authority, and supported by professional associations, both of which have the authority to provide recommendations for clinical practice. Addressing these issues will ultimately lead to improved patient care and outcomes for patients after acquired brain injury.

In conclusion, both standard and non-standard assessment were used to identify vision problems. We found a significant variation in clinical practices among OTs and PTs in Danish hospitals. The results suggest a limited structure and utilization of standardized assessment methods, lack of interdisciplinary collaboration, and an absence of clear clinical guidelines. Furthermore, there seem to be insufficient skills and competence among OTs and PTs in in assessment visual problems.
